# DPA/AA ratio as a potential biomarker of primary aldosteronism in young and middle-aged adults

**DOI:** 10.3389/fendo.2026.1771955

**Published:** 2026-03-11

**Authors:** Wenli Xie, Runlong Lin, Yawen Meng, Lu Zhang, Zhi Zheng, Lijiao Zhang

**Affiliations:** 1 Department of Cardiovascular Medicine, The Second Hospital of Dalian Medical University, Dalian, Liaoning, China; 2 Department of Nuclear Medicine, The Second Hospital of Dalian Medical University, Dalian, Liaoning, China

**Keywords:** arachidonic acid, docosapentaenoic acid, polyunsaturated fatty acid, primary aldosteronism, young and middle-aged adults

## Abstract

**Background:**

Primary aldosteronism (PA) is a common cause of secondary hypertension, yet its diagnosis in young and middle-aged adults remains challenging due to low screening rates and complex testing procedures.

**Objective:**

This study aimed to investigate serum polyunsaturated fatty acid (PUFA) profiles, particularly the docosapentaenoic acid/arachidonic acid (DPA/AA) ratio, as novel biomarkers of PA in young adults.

**Methods:**

This 1:1 matched case–control study was performed on 138 young and middle-aged patients with PA and 138 essential hypertension (EH) controls. Serum PUFA levels were measured, and their associations with PA were analyzed using univariate and multivariate logistic regression analyses. Using bootstrap to conduct internal validation. The receiver operating characteristic (ROC) curve, calibration curve, and decision curve analysis (DCA) were employed to evaluate both the predictive performance and clinical practicability of the model.

**Results:**

Young and middle-aged patients with PA exhibited a distinct phenotype, including more severe hypertension, higher fasting blood glucose level, elevated low-density lipoprotein-cholesterol level, lower platelet count, and greater burden of left ventricular hypertrophy and albuminuria. A significantly reduced DPA/AA ratio was the most notable finding in the PA group. The multivariate analysis revealed DPA/AA as a strong independent inverse predictor of PA. Adding DPA/AA to a baseline model of traditional clinical and biochemical variables increased the area under the curve (AUC) from 0.849 to 0.866. The integration into a more comprehensive model also improved the AUC from 0.872 to 0.887.

**Conclusions:**

The DPA/AA ratio is a robust, independent biomarker of PA in young and middle-aged adults. Its incorporation into diagnostic models significantly enhances predictive accuracy, thus offering a promising noninvasive approach to improve early detection in this high-risk population.

## Introduction

Primary aldosteronism (PA) is a major cause of secondary hypertension, characterized by excessive aldosterone production independently increasing cardiovascular and renal risks beyond the impacts of elevated blood pressure alone (1). The diagnosis of PA is inherently complex, requiring a multistep process of screening, confirmatory testing, with each stage introducing specific interpretive and logistical challenges (2). This complexity is the most crucial bottleneck in timely identification (3). Young and middle-aged patients with hypertension bear a higher lifetime cumulative risk of cardiovascular disease. This risk is profoundly amplified when hypertension is accompanied by metabolic abnormalities, which is a cluster of conditions highly prevalent in patients with PA (4–6). Therefore, diagnosing PA in young and middle-aged individuals is not only challenging but also significantly underprioritized. The systematic screening of young and middle-aged patients with hypertension, especially those presenting with resistant hypertension, hypokalemia, or incidental adrenal nodules, was considered a necessary component of early PA detection strategies. However, a substantial proportion of young and middle-aged patients with PA lack these classic clues (7, 8). Hence, the availability of auxiliary diagnostic tools becomes imperative to identify candidates for PA in this clinically ambiguous yet high-risk subgroup.

Recent studies are exploring diagnostic models for PA integrating traditional clinical and biochemical parameters, such as potassium levels, and aldosterone-to-renin ratio (ARR), to improve diagnostic accuracy (9). Although these markers form a foundational diagnostic framework, their predictive accuracy is suboptimal, especially in young and middle-aged adults. This is often erroneously attributed to lifestyle factors, leading to the under-utilization of ARR screening (10). For example, the widespread use of oral contraceptives and nonsteroidal anti-inflammatory drugs can profoundly alter renin and potassium levels, resulting in misleadingly “normal” ARR values and a high rate of false negatives or false positives (11, 12). Moreover, the definitive but invasive adrenal venous sampling for subtyping is associated with a substantial access barrier (13). This gap underscores the need to discover and validate novel, minimally invasive biomarkers less susceptible to pharmacological interference, thereby enabling timely diagnosis and precision therapy for those who stand to benefit the most.

Marine omega-3 polyunsaturated fatty acids (n-3 PUFAs), primarily eicosapentaenoic acid (EPA), docosapentaenoic acid (DPA), and docosahexaenoic acid (DHA), have been found to be related to the diseases of the cardiovascular system, especially hypertension (14–16). The balance between ω-3 and ω-6 fatty acids is particularly relevant because it may influence key pathophysiological processes such as blood pressure regulation and aldosterone synthesis (17, 18). DHA and EPA, the most studied marine-derived ω-3 PUFAs, exert anti-inflammatory, anti-thrombotic, and vasodilatory effects by modulating membrane fluidity, reducing triglyceride synthesis, and suppressing the expression of pro-inflammatory cytokines (19, 20). In contrast, DPA, the key intermediary in the n-3 metabolic pathway, has been largely overlooked despite increasing evidence of its unique cardioprotective properties (21–25). Limitations such as the low abundance of DPA in conventional fish oils and limited commercial availability have impeded dedicated investigations on this PUFA (26). Its potential role in disease diagnostics should not be overlooked. Given its involvement in inflammatory resolution and metabolic regulation, we hypothesize that DPA and its ratio to the ω-6 fatty acid arachidonic acid (AA) may reflect underlying disease activity, positioning DPA/AA ratio as a potential novel biomarker for improving the early detection and risk stratification of PA.

## Methods

### Study population

According to previous research study (27), the inclusion criteria were as follows: patients aged >18 years, diagnosed with PA using the saline infusion test (SIT) or captopril challenge test (CCT), with results of lipid profiles, glycated hemoglobin (HbA1c), and estimated glomerular filtration rate, and those who had not been taking any lipid-lowering agents, mineralocorticoid antagonists, or amiloride before diagnosis. Patients with severe liver disease (Child-Pugh class ≥B) or uncontrolled hypothyroidism were excluded. Patients with EH, diagnosed based on the current guidelines in China (28), who have aldosterone and renin result, were enrolled as a control group.

### Study participants

This study was performed on 138 young and middle-aged patients with PA at the Second Hospital of Dalian Medical University from January 2019 to December 2022. PA was diagnosed in accordance with the Chinese Medical Association Consensus on the management of PA (29). The diagnosis was based on a positive screening result with the ARR ≥30 ng/dL per ng/mL/h, plus at least one positive result from confirmatory tests including SIT and CCT. Antihypertensive medications were usually changed to calcium channel blockers or alpha blockers until the final diagnosis was made. All diagnosed patients underwent computed tomography scanning. Young and middle-aged patients with PA were defined as ≤55 or ≤65 years of age in men or women, respectively (30). Hospitalized age-matched individuals with EH during the same period were screened and included as the control group.

**Figure 1:**
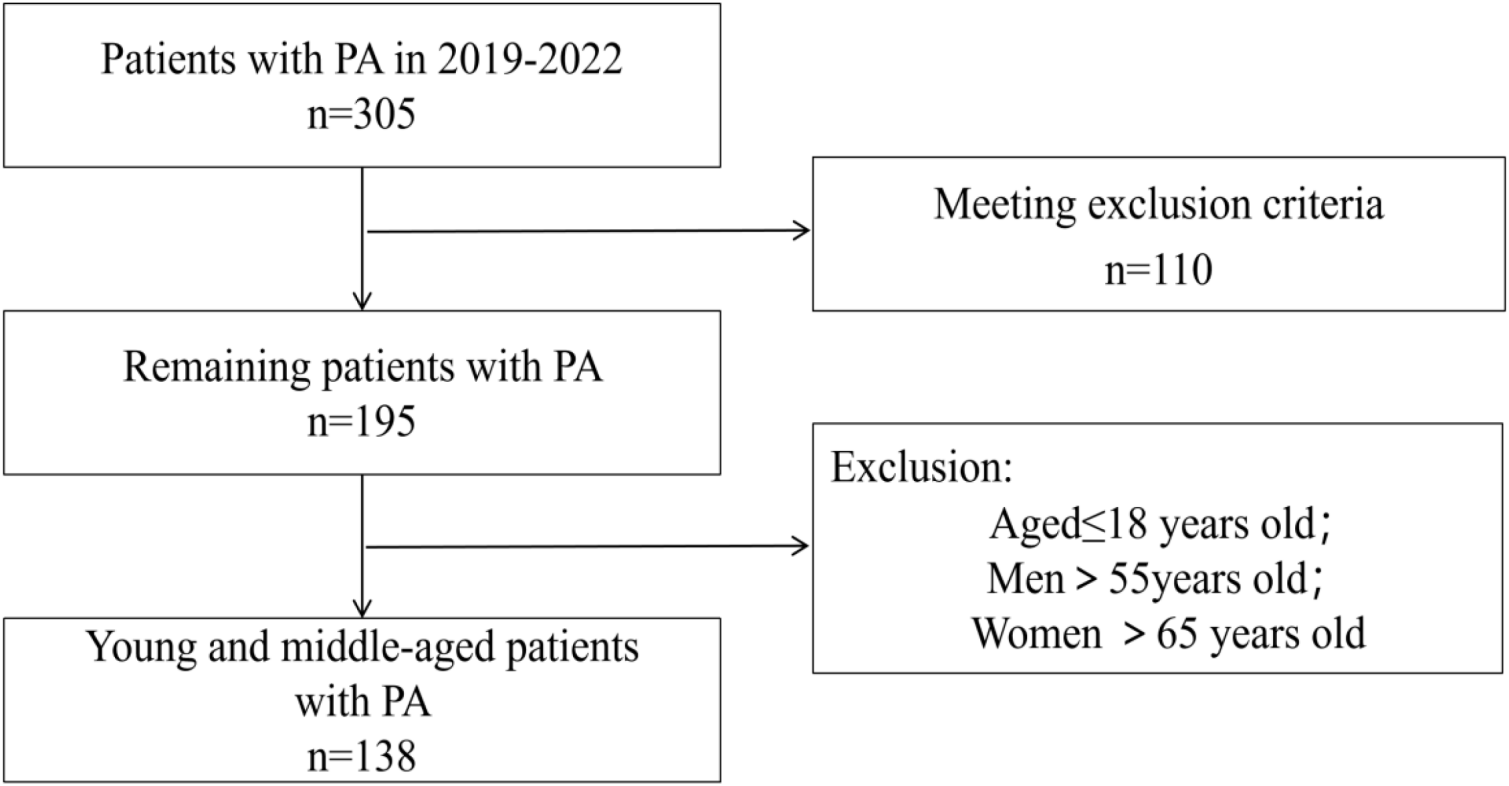
Flow diagram for patient selection.

The exclusion criteria were as follows: (1) secondary hypertension other than PA; (2) consumption of fish oil or PUFA supplement in the last 3 months; (3) uncontrolled infectious disease, autoimmune disease, end-stage renal disease, acute hepatitis, psychiatric disorders, moderate-to-severe anemia, uncontrolled hyperthyroidism and hypothyroidism, or malignant tumor in the last 3 months; (4) diagnosis of uncontrolled heart failure (New York Heart Association Grades III and IV), unstable angina, myocardial infarction, sick sinus syndrome, persistent atrial fibrillation, pulmonary heart disease, acute and chronic pulmonary infection, congenital heart disease, and rheumatic valvular heart disease; (5) previous usage of lipid-lowering agents, mineralocorticoid antagonists, or amiloride; (6) drug abuse, cachexia; (7) lack of baseline data. The enrolled patients are shown in the flow diagram, show in **Figure 1**.

### Collection of clinical data

The clinical data of patients were collected from medical records, including age, sex, body mass index, and history of diabetes mellitus (DM), hyperlipidemia, hyperuricemia, obesity, systolic blood pressure (SBP), and diastolic blood pressure (DBP). Dyslipidemia was determined with reference to current Chinese prevention and treatment guidelines for dyslipidemia (31). It was defined as meeting any of the following criteria: total cholesterol (TC) ≥6.2 mmol/L, triglyceride (TG) ≥2.3 mmol/L, high-density lipoprotein-cholesterol (HDL-C) <1.0 mmol/L, and low-density lipoprotein-cholesterol (LDL-C) ≥4.1 mmol/L. Type 2 diabetes mellitus was defined as a fasting blood glucose (FBG) level of ≥7.0 mmol/L, or glycosylated hemoglobin of ≥6.5%, or treatment with hypoglycemic medications (32). Obesity was defined as a BMI ≥28 kg/m^2^ in accordance with the guidelines for the management of obesity in China (33). We also evaluated the following laboratory parameters: white blood cell (WBC) count, hemoglobin (HGB) level, platelet (PLT) count, and TC, TG, HDL-C, LDL-C, blood potassium, blood sodium, FBG, creatinine, uric acid (UA), alanine aminotransferase (ALT), aspartate aminotransferase (AST), urine microalbumin (UMA), urine creatinine, and urine microalbumin/creatinine (UACR).

Additionally, we collected echocardiography results, including left atrial diameter, left ventricular end-diastolic diameter (LVDD), interventricular septum (IVS), left ventricular posterior wall (LVPW), and calculated left ventricular mass (LVM). Left ventricular mass index (LVMI) was the ratio of LVM to body surface area (BSA). LVM (g) = 0.8 × 1.04 × [(IVS + LVPW + LVDD)³ – LVDD³] + 0.6; BSA (m²) = 0.0061 × height (cm) + 0.0128 × weight (kg) – 0.1529; LVMI (g/m²) = LVM (g)/BSA (m²). The criteria for left ventricular hypertrophy were LVMI >115 g/m² in men and >95 g/m²in women, or left ventricular wall thickness (LVWT) ≥12 mm (34).

### Measurement of the serum levels of PUFAs and other laboratory analyses

All enrolled patients had fasting blood samples collected after 8 h of fasting on the morning of the day after admission. The PUFA levels were determined using a isotope dilution-liquid chromatography–tandem mass spectrometry system (AB SCIEX Triple Quad 4500MD LC-MS/MS), including EPA, DHA, DPA, and AA. The analytical method employed in this study has a strict operational procedure and good reproducibility with identical sample sequences. The detailed protocol is provided in the **Supplementary Materials**. The ratios of PUFA levels were computed, for example, EPA/AA was the ratio of EPA to AA, DPA/AA was the ratio of DPA/AA, DHA/AA was the ratio of DHA to AA, (EPA + DHA)/AA was the ratio of the sum of EPA and DHA to AA, (EPA + DPA)/AA was the ratio of EPA and DPA to AA, (DHA + DPA)/AA was the ratio of the sum of DHA and DPA to AA, (EPA + DHA + DPA)/AA was the ratio of the sum of EPA, DHA, and DPA to AA.

### Statistical analysis

The data were analyzed using SPSS Statistics 26.0 and R 4.2.3. Continuous variables were tested for normality using the Kolmogorov–Smirnov test. Normally distributed variables were expressed as mean ± standard deviation (X ± S), non-normally distributed variables were expressed as median (P25, P75), and categorical variables were expressed as number and percentage (%). Differences between two independent samples were analyzed using the *t* test or Mann–Whitney *U* test. The ggplot2 package was used for box plot and bean plot visualization. The *χ*^2^ test was used to compare rates between two independent samples. Multivariate logistic regression analysis (stepwise method) was used to establish a model for variable selection and intergroup predictive factors. Forest plots were drawn using R software to visualize the risk factors. Indicators with a *P* value less than 0.05 were included in the analyses and plotted on a receiver operating characteristic (ROC) graph, with the area under the curve (AUC) measured by the C-statistic used to quantify predictive power. Internal validation of the diagnostic models was conducted using the bootstrap method with 1000 resamples to assess model robustness and reduce overfitting bias. Calibration curves and decision curve analysis (DCA) was employed to evaluate the performance and clinical utility of these models by estimating net benefits at various threshold probabilities. *P* value less than 0.05 indicated a statistically significant difference.

## Results

### Demographic and clinical characteristics

Comparisons of patients with PA and EH are presented in **Table 1**. We conducted a 1:1 match of 138 patients with PA to an equal number of patients with EH by sex, age, and BMI. Patients with PA had significantly higher SBP, DBP, FBG, blood sodium, LDL-C and DM prevalence (*P* < 0.05), but lower PLT, blood potassium, UA, ALT, and AST than EH patients (*P* < 0.05). Regarding the target organ damage, left ventricular hypertrophy was more frequent in the PA group. Additionally, UACR was significantly higher in the PA group than in the EH group. Collectively, these distinct disparities in clinical, biochemical, and target organ damage-related parameters may serve as valuable indicators to facilitate the differential inference of primary aldosteronism (PA).

**Table 1:** Demographic and clinical characteristics of young and middle-aged patients with PA.

Characteristic	Patients with EH (*n* = 138)	Patients with PA (*n* = 138)	*P* value
Demographic characteristics			
Age, year	52.67 ± 10.10	51.58 ± 9.40	0.356
Male, *n* (%)	71 (51.40)	79 (57.20)	0.334
BMI, kg/m^2^	27.20 ± 4.02	26.63 ± 4.33	0.262
SBP, mm Hg	145.02 ± 19.90	156.83 ± 24.26	<0.001
DBP, mm Hg	93.15 ± 13.28	98.83 ± 15.02	<0.001
DM, *n* (%)	22 (15.90)	40 (29.00)	0.009
Hyperlipidemia, *n* (%)	49 (35.50)	62 (44.90)	0.111
Hyperuricemia, *n* (%)	62 (44.90)	52 (37.70)	0.222
Obesity, *n* (%)	52 (37.70)	45 (32.60)	0.377
Biochemical indices			
WBC count, ×10^9^/L	6.52 ± 1.82	6.44 ± 1.83	0.723
HGB, g/L	142.77 ± 19.03	140.42 ± 15.75	0.267
PLT count, ×10^9^/L	244.23 ± 61.79	228.96 ± 54.67	0.031
Blood potassium, mmol/L	4.06 ± 0.35	3.49 ± 0.57	<0.001
Blood sodium, mmol/L	141.35 ± 1.92	142.23 ± 2.44	0.001
FBG, mmol/L	5.80 ± 1.36	6.29 ± 1.84	0.013
TC, mmol/L	4.77 ± 0.89	4.85 ± 0.88	0.420
TG, mmol/L	1.85 ± 1.10	1.89 ± 1.15	0.751
HDL-C, mmol/L	1.21 ± 0.29	1.17 ± 0.31	0.365
LDL-C, mmol/L	2.47 ± 0.66	2.72 ± 0.64	0.001
Scr, µmol/L	67.48 ± 23.39	73.18 ± 32.37	0.097
UA, µmol/L	361.24 (305.68–447.74)	342.64(283.53–407.69)	0.027
ALT, U/L	23.42 (17.20–34.80)	19.73 (14.52–29.10)	0.006
AST, U/L	21.24 (17.54–26.85)	18.28 (15.19–23.02)	<0.001
Alb,g/L	43.21 ± 3.37	42.48 ± 3.60	0.084
Target organ damage			
Left ventricular hypertrophy, *n* (%)	27 (19.60)	46 (33.30)	0.010
UACR, mg/g	15.53 (9.82–27.27)	25.79 (10.89–70.45)	0.005

Data are presented as percentages, mean and SD, and median and interquartile range. ALT, Alanine aminotransferase; AST, aspartate aminotransferase; BMI, body mass index; DBP, diastolic blood pressure; DM, diabetes mellitus; EH, essential hypertension; FBG, fasting blood glucose; HDL-C, high-density lipoprotein-cholesterol; HGB, hemoglobin; LDL-C, low-density lipoprotein-cholesterol; PA, primary aldosteronism; PLT, platelets; SBP, systolic blood pressure; Scr, serum creatinine; TC, total cholesterol; TG, triglyceride; UA, uric acid; UACR, urine albumin creatine ratio; WBC, white blood cell.

### Comparison of ω-3, ω-6 PUFAs and ratios of ω-3/ω-6 PUFAs of PA versus EH in young and middle-aged adults

**Figure 2:**
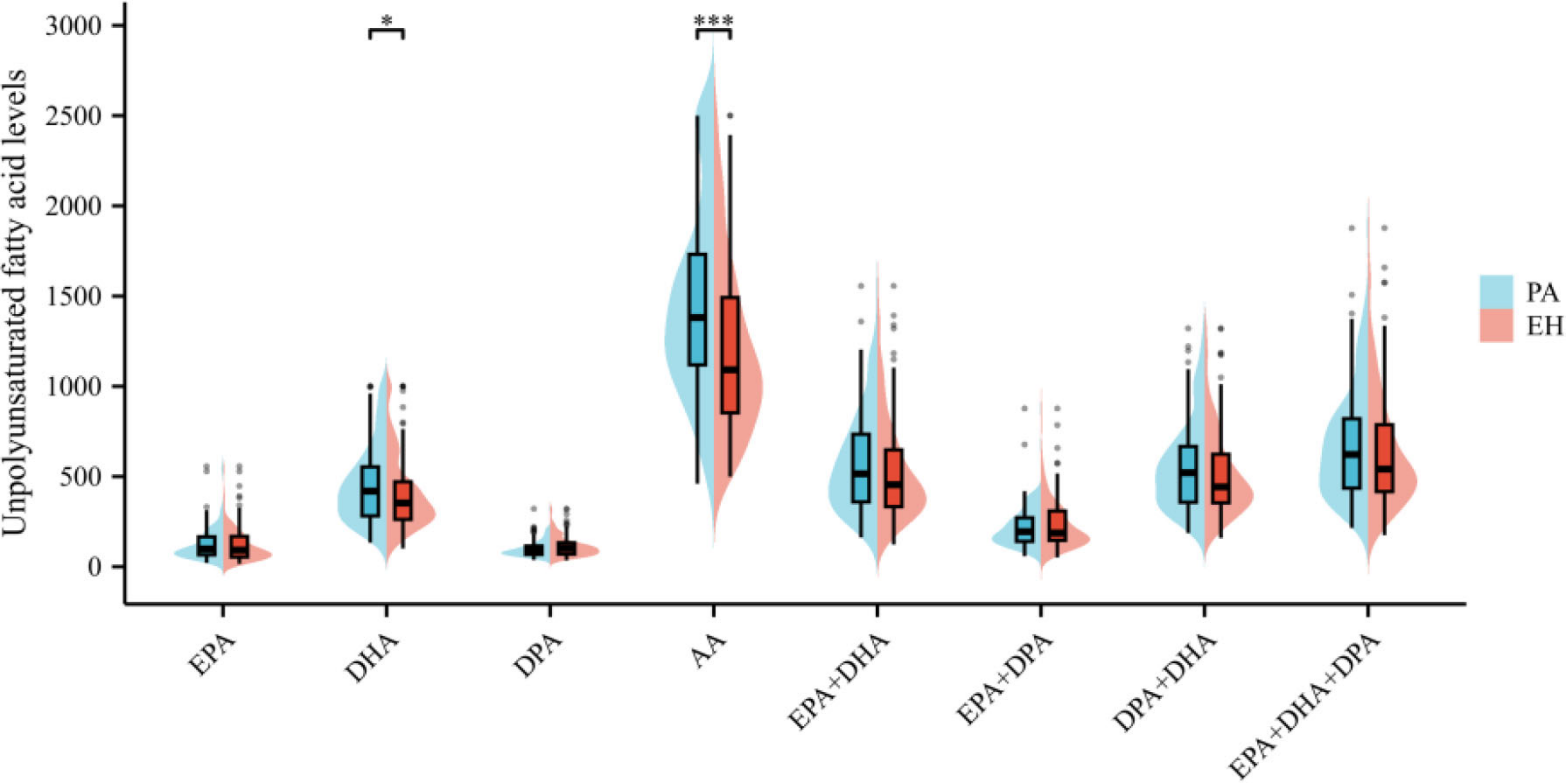
Comparison of each polyunsaturated fatty acid level in young patients with PA. The levels of DHA and AA were significantly higher in patients with PA than in patients with EH. However, the levels of EPA, DPA, EPA + DHA, EPA + DPA, DPA + DHA, and EPA + DHA + DPA showed no statistically significant differences. ^*^P < 0.05; ^***^P < 0.001.

**Figure 3:**
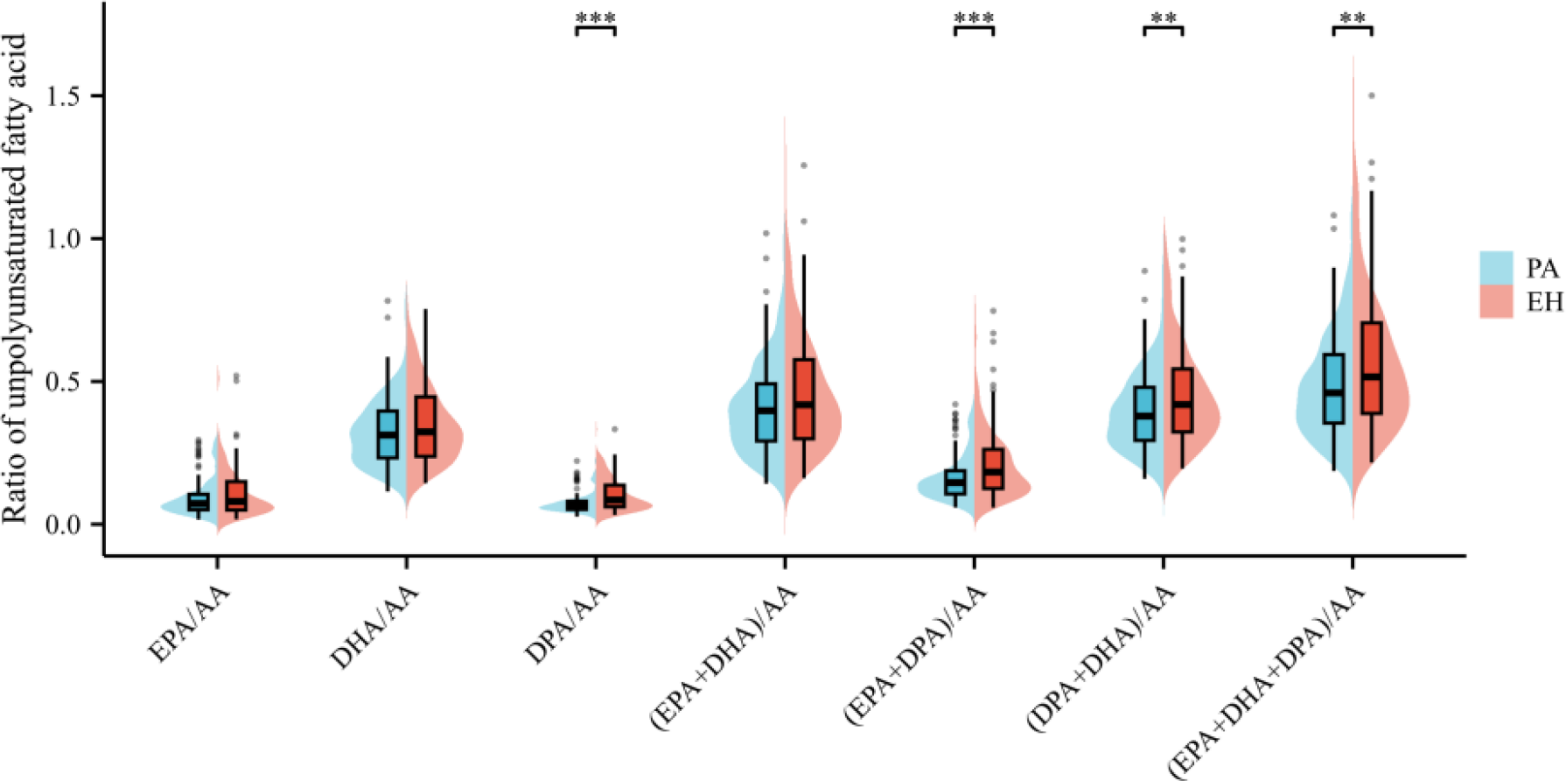
Comparison of the ratio of polyunsaturated fatty acids in young patients with PA. The ratio of DPA/AA, (EPA + DPA)/AA, (DPA + DHA)/AA, and (EPA + DHA + DPA)/AA were also reduced in patients with PA than in those with EH. No significant difference was observed in EPA/AA, DHA/AA, and (EPA + DHA)/AA ratios. ^**^P < 0.01; ^***^P < 0. 001.

The distribution of serum ω-3, ω-6 PUFAs, along with their ratios, differed between patients with PA and those with EH. **Figure 2** visually summarizes these differences using bean plots, and the specific data are presented in **Supplementary Table 1**. In stark contrast, distinct disparities were observed in the ratios of ω-3/ω-6 PUFAs between the two groups, particularly for ratios incorporating DPA. As shown in **Figure 3**, the ratio of DPA/AA was lower in the PA group than in the EH group [median (IQR): 0.08 (0.06–0.14) vs 0.06 (0.05–0.08); *P* < 0.01]. Similarly, the ratios of (EPA + DPA)/AA, (DPA + DHA)/AA, and (EPA + DHA + DPA)/AA were also reduced in patients with PA than in those with EH. These differences prompted further investigation into their associations with PA risk via regression analyses.

### Univariate regression analysis of the association between ω-3, ω-6 PUFAs, ω-3/ω-6 PUFA ratios and PA in young and middle-aged patients

We investigated the association between PUFAs and PA using univariate logistic regression analysis. As shown in **Table 2**, the most pronounced effects were observed for their ratios to AA. EPA/AA (OR = 0.02, 95% CI: 0.010–0.683, *P* < 0.001), DHA/AA (OR = 0.138, 95% CI: 0.019–0.995, *P* = 0.028), DPA/AA (OR = 0.001, 95% CI: 0.000–0.001, *P* < 0.001) were associated with an extraordinarily reduced odds of PA. Similarly, the combined ratio of (EPA + DHA)/AA (OR = 0.220, 95% CI: 0.057–0.851, *P* = 0.028), (EPA + DPA)/AA (OR = 0.004, 95% CI: 0.000–0.069, *P* < 0.001), (DHA + DPA)/AA (OR = 0.068, 95% CI: 013–0.352, *P* = 0.001), (EPA + DHA + DPA)/AA (OR = 0.153, 95% CI: 0.046–0.508, *P* = 0.002) were also inversely associated with the odds of PA. These univariate associations prompted subsequent multivariate logistic regression to validate the independent predictive value of PUFA/AA ratios for PA, adjusted for traditional clinical and biochemical variables.

**Table 2:** Univariate regression analysis of ω-3, ω-6 PUFAs and ratios of ω-3/ω-6 PUFAs in young and middle-aged patients with PA.

Fatty acids (nmol/mL)	*β* value	*P* value	Exp(B)	95% CI	
EPA	0.000	0.932	1.000	0.997	1.002
DHA	0.001	0.037	1.001	1.000	1.002
DPA	–0.005	0.031	0.995	0.990	1.000
AA	0.001	<0.001	1.001	1.001	1.002
EPA + DHA	0.001	0.125	1.001	1.000	1.002
EPA + DPA	–0.001	0.345	0.999	0.997	1.001
DHA + DPA	0.001	0.180	1.001	1.000	1.002
EPA + DHA + DPA	0.000	0.312	1.000	1.000	1.001
EPA/AA	–3.929	0.030	0.020	0.010	0.683
DHA/AA	–1.977	0.039	0.138	0.019	0.995
DPA/AA	–17.180	<0.001	0.001	0.000	0.001
(EPA + DHA)/AA	–1.515	0.028	0.220	0.057	0.851
(EPA + DPA)/AA	–5.425	<0.001	0.004	0.000	0.069
(DHA + DPA)/AA	–2.685	0.001	0.068	0.013	0.352
(EPA + DHA + DPA)/AA	–1.880	0.002	0.153	0.046	0.508

### Multivariate regression analysis of traditional clinical variables in distinguishing PA from EH in young and middle-aged patients

**Figure 4:**
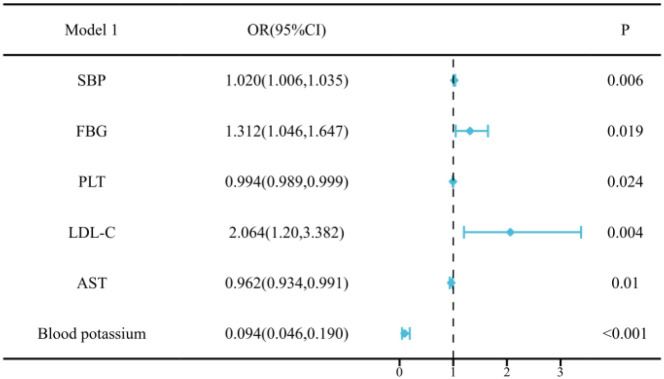
Multivariate regression analysis of Model 1 of PA in young and middle-aged patients. Model 1 included SBP, FBG, PLT, LDL-C, AST, and potassium levels. Multivariate regression analysis demonstrated that SBP, FBG, and LDL-C were significantly associated with an increased risk of PA, whereas PLT, AST, and blood potassium were inversely associated with PA.

**Figure 5:**
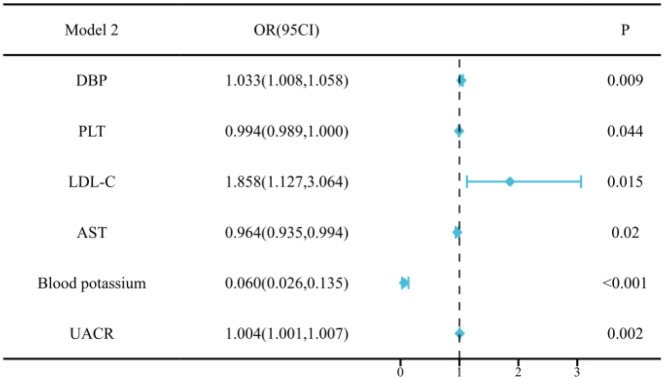
Multivariate regression analysis of Model 2 of PA in young and middle-aged patients. Model 2 additionally included left ventricular hypertrophy and UACR. Multivariate regression analysis indicated that DBP, LDL-C, and UACR emerged as significant positive predictors. Blood potassium remained a strong inverse predictor.

We conducted multivariate logistic regression analysis and constructed two models to identify independent factors associated with PA. The results of both models are summarized in **Supplementary Table 2** and presented visually as forest plots in **Figures 4**, **5**.

The analysis of Model 1 revealed that SBP, FBG, and LDL-C were significantly associated with an increased risk of PA,while lower blood potassium was a strong inverse predictor.

Model 2 additionally included left ventricular hypertrophy and UACR. After adjustment, DBP, LDL-C, and UACR emerged as significant positive predictors. And lower blood potassium remained the most prominent inverse predictor of PA. Detailed results for all variables are provided in the **Supplementary Table 2**.

### Multivariate regression analysis of ω-3, ω-6 PUFAs, ω-3/ω-6 PUFA ratios and traditional clinical variables for predicting PA in young and middle-aged patients

Multivariate logistic regression analyses were performed to evaluate associations between various PUFA ratios and the risk of PA in young and middle-aged patients. Model 1 adjusted for SBP, DBP, blood potassium, PLT, FBG, ALT, AST, LDL-C, and UA. Model 2 adjusted for the variables of Model 1 and left ventricular hypertrophy and UACR.

As shown in **Table 3**, most PUFA ratios demonstrated significant inverse associations with PA whether adjusted for traditional cardiovascular risk factors or not. The ratio of DPA/AA was significantly associated with a reduced risk of PA in both Model 1 (OR = 0.001, 95% CI: 0.000–0.001, *P* < 0.001) and Model 2 (OR = 0.001, 95% CI: 0.000–0.001, *P* < 0.001), indicating its potent protective effect. Similarly, the combined ratios of (EPA + DPA)/AA, (DHA + DPA)/AA, and (EPA + DHA + DPA)/AA also demonstrated significant inverse associations in both Model 1 and Model 2.

**Table 3:** Multivariate regression analysis of PUFAs and the traditional risk factors of PA in young and middle-aged patients.

Index	Model 1		Model 2	
	OR (95% CI)	*P*	OR (95% CI)	*P*
EPA/AA	0.205 (0.002–21.518)	0.504	0.126 (0.001–16.730)	0.406
DHA/AA	0.098 (0.007–0.135)	0.083	0.094 (0.006–1.570)	0.100
DPA/AA	0.001 (0.000–0.001)	<0.001	0.001 (0.000–0.001)	<0.001
(EPA + DHA)/AA	0.252 (0.040–1.564)	0.139	0.234 (0.034–1.626)	0.142
(EPA + DPA)/AA	0.020 (0.001–0.690)	0.030	0.018 (0.000–0.769)	0.036
(DHA + DPA)/AA	0.062 (0.007–0.565)	0.014	0.061 (0.006–0.654)	0.021
(EPA + DHA + DPA)/AA	0.186 (0.037–0.943)	0.042	0.172 (0.030–0.977)	0.047

Model 1 included SBP, DBP, blood potassium, blood sodium, PLT, FBG, ALT, AST, LDL-C, and UA. Model 2 included the variables of model 1 and left ventricular hypertrophy and UACR.

### Clinical use

**Figure 6:**
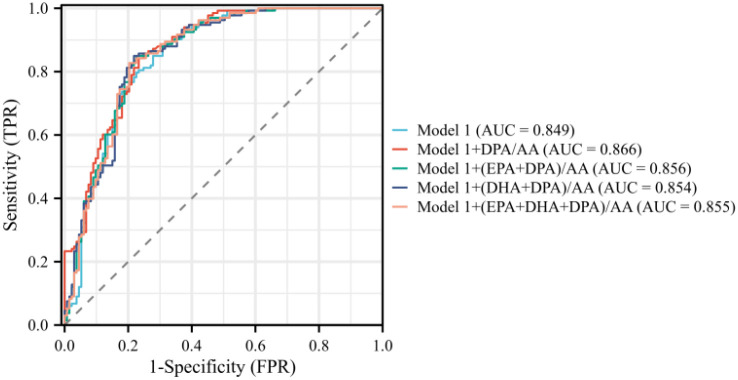
ROC curves for Model 1 and PUFAs of PA in young and middle-aged patients.

**Figure 7:**
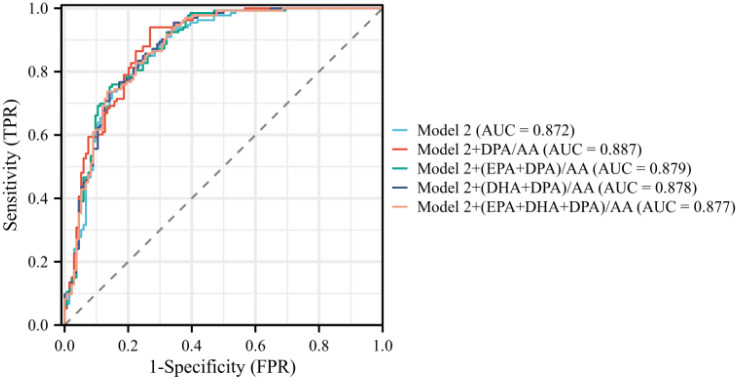
ROC curves for Model 2 and PUFAs of PA in young and middle-aged patients.

ROC curve analysis was performed to evaluate the discriminative utility of Model 1 and Model 2 (seen in **Figures 6**, **7**), both alone and in combination with various PUFA ratios. For young PA patients, Model 1 showed good discriminative ability with an AUC of 0.849, and incorporating PUFA ratios further improved its performance; Model 1 + DPA/AA achieved the optimal enhancement with the highest AUC of 0.866. Model 2, incorporating traditional variables, left ventricular hypertrophy and UACR, exhibited an AUC of 0.872,with Model 2 + DPA/AA again delivering the most notable improvement to an AUC of 0.887. Bootstrap internal validation, calibration curves, and decision curve analysis (DCA) supported the reliability and clinical utility of all models. Detailed validation figures are provided in **Supplementary Figures 1-3**.

## Discussion

Although the latest clinical guidelines recommend PA screening for all hypertensive patients, primary aldosteronism (PA) remains clinically challenging to diagnose due to its complex pathogenesis and multiple confounding factors; notably, the integration of novel diagnostic biomarkers is imperative to improve PA diagnostic efficiency (35). Our case–control study yielded several pivotal findings regarding the diagnosis of PA in young and middle-aged adults. First, we confirmed and extended the clinical phenotype of young and middle-aged patients with PA, demonstrating not only more severe hypertension but also a significant metabolic and PLT derangement. Specifically, these patients presented with higher FBG and elevated LDL-C levels. The dyslipidemia observed might be directly mediated by aldosterone, which upregulates hepatic lipid synthesis and impairs clearance, thereby contributing to atherogenic risk (4). This metabolic profile was consistent with the known interplay between aldosterone excess and metabolic syndrome, which might exacerbate cardiovascular risk (36). Furthermore, we observed a lower PLT count in the PA group. This was a finding previously linked to aldosterone-driven pro-inflammatory and profibrotic states, potentially reflecting a higher degree of endothelial activation and subclinical vascular damage (14, 37–39).

This phenotype was coupled with a greater burden of subclinical cardiac and renal damage compared with their EH counterparts. The cardiac remodeling, primarily left ventricular hypertrophy, is a direct consequence of the profibrotic and inflammatory effects of aldosterone on the myocardium, independent of blood pressure (1). Similarly, renal damage, which is manifested as elevated UACR, results from aldosterone-mediated glomerular and tubular injury, which promotes proteinuria and fibrosis (40).

Critically, the convergence of severe hypertension, metabolic dysregulation, and early subclinical organ damage in young and middle-aged patients with PA portends a uniquely adverse long-term prognosis. Young-onset PA is associated with a significantly higher risk of cardiovascular events and irreversible organ dysfunction compared with that in age-matched patients with EH, underscoring the aggressive nature of the disease in this demographic and the imperative for early diagnosis (41).

The demographic and clinical characteristics observed in our PA cohort aligned with the known detrimental effects of chronic aldosterone excess. The significantly lower serum potassium levels and higher blood pressure were hallmark features. The increased incidence of left ventricular hypertrophy and higher UACR underscored the accelerated target organ damage in young and middle-aged patients with PA, reinforcing the imperative for early diagnosis and intervention. Beyond these classic signs, our findings revealed a significant metabolic dimension to PA. The association of higher LDL-C and FBG with PA further suggested a complex interplay between aldosteronism and metabolic dysregulation. This notion is supported by a growing body of evidence. For instance, studies have indicated a bidirectional relationship between blood glucose level and PA, where insulin resistance may exacerbate aldosterone production and vice versa (42–45). Similarly, the alterations in lipid metabolism have been implicated in PA pathogenesis, with certain lipids potentially influencing adrenal steroidogenesis (4, 46, 47). This intertwined pathophysiology may exacerbate long-term cardiovascular risk in patients. Beyond being mere comorbidities, these metabolic disturbances represent promising auxiliary biomarkers that can enhance early diagnostic strategies for PA. Their integration into diagnostic models can enhance the identification of PA, particularly in young and middle-aged individuals, where traditional signs alone may not suffice.

The core of our investigation revolved around the potential of PUFA ratios as novel biomarkers. Although individual PUFA levels showed differences, their diagnostic power was vastly superior when expressed as ratios to AA, particularly for the ω-3 PUFAs. This aligned with the increasing understanding that the balance between pro-inflammatory ω-6 and anti-inflammatory ω-3 PUFAs is more physiologically relevant than their absolute levels (20, 48). The most striking finding was the extraordinary inverse association between the DPA/AA ratio and PA (49, 50). The remarkably low odds ratios (OR = 0.001) in our multivariable models suggested a lower DPA/AA ratio as an exceptionally strong indicator of PA, independent of traditional risk factors such as blood pressure and potassium.

The superior performance of DPA/AA, compared with the more commonly studied EPA/AA and DHA/AA, prompts speculation on its potential mechanistic role. DPA is not merely an intermediate between EPA and DHA; it possesses unique biological activities, including potent anti-inflammatory effects via specialized pro-resolving mediators (SPMs) (14, 49). We hypothesize that the profoundly low DPA/AA ratio in PA may reflect one of two pathophysiological states: either an increased consumption or use of DPA in an attempt to counterbalance the pro-inflammatory and profibrotic state induced by aldosterone excess, or a fundamental alteration in PUFA metabolism driven by the disease itself, potentially at the level of elongase or desaturase enzymes, as hinted recently. In essence, this imbalance represents a state where DPA use exceeds its biosynthesis and supply (18, 51). This disruption in the PUFAs may directly contribute to the critical resolution deficit: a specific shortage of DPA-derived SPMs needs to quench the chronic sterile inflammation characterizing PA. This deficit not only permits sustained inflammation but also directly enables the progression of the dysfunction of the endothelial cells, smooth muscle cells, and PLT observed in PA. This leads to an irreversible maladaptive remodeling in the heart and vessels (37, 52, 53). Therefore, measuring the DPA/AA ratio provides a window into this active disease process. Its value extends beyond diagnosis; quantification of this resolution deficit may serve as a direct readout of the biological burden driving target organ damage (e.g., left ventricular hypertrophy and albuminuria), thereby offering a novel biomarker for risk stratification and prognostication (54).

From a clinical standpoint, the incremental predictive value of the DPA/AA ratio is highly significant. The ROC analyses showed that adding DPA/AA to a model of traditional clinical variables (Model 1) increased the AUC from 0.849 to 0.866. Even when the model was already strengthened by including the markers of target organ damage (Model 2, AUC = 0.872), the DPA/AA ratio provided a further, albeit smaller, improvement (AUC = 0.887). This demonstrated that the DPA/AA ratio provided unique diagnostic information not captured by current standard clinical parameters. As a serum biomarker less susceptible to clinical interference, the DPA/AA ratio can serve as a more stable and reliable screening tool, prompting further investigation in individuals where ARR is equivocal or unreliable.

## Limitations and future directions

Our study had several limitations to be considered. First, its case–control design inherently prevented the inference of causality, leaving the temporal relationship between the altered PUFA profile and PA onset unresolved. Second, the single-center nature and relatively modest sample size, though sufficient for initial discovery, warrant external validation in larger, prospective, and multi-ethnic cohorts to ensure generalizability. A significant limitation was the lack of data on PA subtypes (e.g., unilateral aldosterone-producing adenoma vs bilateral adrenal hyperplasia), precluding an analysis of whether the observed lipidomic alterations, particularly the DPA/AA ratio, differ across subtypes or are associated with the success of specific treatments such as adrenalectomy. Furthermore, we measured the levels of total serum PUFAs, which, though accessible, might not fully reflect the biologically active composition within critical target tissues such as the adrenal gland, adipose tissue, or cellular membranes.

Future research based on these findings should focus on several key areas: (1) prospectively validating the DPA/AA ratio as a diagnostic and risk-stratification tool in independent cohorts of young and middle-aged patients with hypertension; (2) investigating the precise molecular mechanisms (potentially involving altered elongase/desaturase activities or inflammatory resolution pathways) linking aldosterone excess to DPA/AA dysregulation; (3) examining the utility of the DPA/AA ratio in differentiating PA subtypes and its potential to predict therapeutic response and long-term prognosis, thereby refining personalized treatment strategies; and (4) exploring whether targeted ω-3 PUFA supplementation, particularly with DPA, can modulate disease activity, reverse the lipidomic imbalance, or improve clinical outcomes in patients with PA.

## Conclusions

In summary, our study identified the DPA/AA ratio as a robust, independent biomarker that significantly enhances the discriminative performance of both traditional risk factor-based (Model 1) and target organ damage-enriched (Model 2) predictive models for young and middle-aged PA patients. Integrating this ratio into models based on conventional factors significantly enhanced their accuracy, meeting the immediate need for better noninvasive tools in a population where early detection was crucial. These results not only advance clinical risk assessment but also open new research directions into how PUFA dysregulation contributes to this disease.

## Data Availability

The original contributions presented in the study are included in the article/**Supplementary Material**. Further inquiries can be directed to the corresponding author.

## References

[1] Monticone S D'Ascenzo F Moretti C Williams TA Veglio F Gaita F . Cardiovascular events and target organ damage in primary aldosteronism compared with essential hypertension: A systematic review and meta-analysis. Lancet Diabetes Endocrinol. (2018) 6:41–50. doi: 10.1016/S2213-8587(17)30319-4, PMID: 29129575

[2] Funder JW Carey RM . Primary aldosteronism: where are we now? Where to from here. Hypertension. (2022) 79:726–35. doi: 10.1161/HYPERTENSIONAHA.121.18761, PMID: 35067069

[3] Zhang Y Vittinghoff E Pletcher MJ Allen NB Zeki Al Hazzouri A Yaffe K . Associations of blood pressure and cholesterol levels during young adulthood with later cardiovascular events. J Of Am Coll Of Cardiol. (2019) 74:330–41. doi: 10.1016/j.jacc.2019.03.529, PMID: 31319915 PMC6764095

[4] Liang NP Rao KR Hu M Bao RY Liu JW Lai ZQ . The association between aldosterone and lipid profiles in patients with primary aldosteronism. Sci Rep. (2025) 15:8755. doi: 10.1038/s41598-025-92477-9, PMID: 40082504 PMC11906652

[5] Colussi G Catena C Lapenna R Nadalini E Chiuch A Sechi LA . Insulin resistance and hyperinsulinemia are related to plasma aldosterone levels in hypertensive patients. Diabetes Care. (2007) 30:2349–54. doi: 10.2337/dc07-0525, PMID: 17575088

[6] Steichen O . Primary aldosteronism and diabetes mellitus. Horm Metab Res. (2010) 42:758–9; author reply 760. doi: 10.1055/s-0030-1261966, PMID: 20648412

[7] Funder JW Carey RM Mantero F Murad MH Reincke M Shibata H . The management of primary aldosteronism: Case detection, diagnosis, and treatment: an endocrine society clinical practice guideline. J Clin Endocrinol Metab. (2016) 101:1889–916. doi: 10.1210/jc.2015-4061, PMID: 26934393

[8] Käyser SC Dekkers T Groenewoud HJ van der Wilt GJ Carel Bakx J van der Wel MC . Study heterogeneity and estimation of prevalence of primary aldosteronism: A systematic review and meta-regression analysis. J Clin Endocrinol Metab. (2016) 101:2826–35. doi: 10.1210/jc.2016-1472, PMID: 27172433

[9] Manosroi W Tacharearnmuang N Atthakomol P . Clinical and biochemical predictors and predictive model of primary aldosteronism. PloS One. (2022) 17:e0272049. doi: 10.1371/journal.pone.0272049, PMID: 35930535 PMC9355203

[10] Bruno RM Pucci G Rosticci M Guarino L Guglielmo C Agabiti Rosei C . Association between lifestyle and systemic arterial hypertension in young adults: A national, survey-based, cross-sectional study. High Blood Pressure Cardiovasc Prev. (2016) 23:31–40. doi: 10.1007/s40292-016-0135-6, PMID: 26909755

[11] Rossi GP Rossi FB Guarnieri C Rossitto G Seccia TM . Clinical management of primary aldosteronism: An update. Hypertension. (2024) 81:1845–56. doi: 10.1161/HYPERTENSIONAHA.124.22642, PMID: 39045687

[12] Pizzolo F Raffaelli R Memmo A Chiecchi L Pavan C Guarini P . Effects of female sex hormones and contraceptive pill on the diagnostic work-up for primary aldosteronism. J Hypertens. (2010) 28:135–42. doi: 10.1097/HJH.0b013e32833266e3, PMID: 19952782

[13] Gkaniatsa E Sakinis A Palmér M Muth A Trimpou P Ragnarsson O . Adrenal venous sampling in young patients with primary aldosteronism. Extravagance or irreplaceable. J Clin Endocrinol Metab. (2021) 106:e2087–95. doi: 10.1210/clinem/dgab047, PMID: 33507307

[14] Saravanan P Davidson NC Schmidt EB Calder PC . Cardiovascular effects of marine omega-3 fatty acids. Lancet. (9740) 20102025. 376:540–50. doi: 10.1016/s0140-6736(10)60445-x, PMID: 20638121

[15] Geleijnse JM Giltay EJ Grobbee DE Donders ART Kok FJ . Blood pressure response to fish oil supplementation: Metaregression analysis of randomized trials. J Of Hypertens. (2002) 20:1493–9. doi: 10.1097/00004872-200208000-00010, PMID: 12172309

[16] Morris MC Sacks F Rosner B . Does fish oil lower blood pressure? A meta-analysis of controlled trials. Circulation. (1993) 88:523–33. doi: 10.1161/01.CIR.88.2.523, PMID: 8339414

[17] Das UN . Essential fatty acids and their metabolites could function as endogenous HMG-CoA reductase and ACE enzyme inhibitors, anti-arrhythmic, anti-hypertensive, anti-atherosclerotic, anti-inflammatory, cytoprotective, and cardioprotective molecules. Lipids Health Dis. (2008) 7:37. doi: 10.1186/1476-511X-7-37, PMID: 18922179 PMC2576273

[18] Ling G Bruno J Albert SG Dhindsa S . Fatty acids as a direct regulator of aldosterone hypersecretion. Mol And Cell Endocrinol. (2023) 561:111836. doi: 10.1016/j.mce.2022.111836, PMID: 36549461

[19] Li Y Tang H Yang X Ma L Zhou H Zhang G . Associations of ω-3, ω-6 polyunsaturated fatty acids intake and ω-6: ω-3 ratio with systemic immune and inflammatory biomarkers: NHANES 1999-2020. Front Nutr. (2024) 11. doi: 10.3389/fnut.2024.1410154, PMID: 38912301 PMC11190316

[20] Tortosa-Caparrós E Navas-Carrillo D Marín F Orenes-Piñero E . Anti-inflammatory effects of omega 3 and omega 6 polyunsaturated fatty acids in cardiovascular disease and metabolic syndrome. Crit Rev In Food Sci And Nutr. (2017) 57:3421–9. doi: 10.1080/10408398.2015.1126549, PMID: 26745681

[21] Harris WS Del Gobbo L Tintle NL . The Omega-3 Index and relative risk for coronary heart disease mortality: Estimation from 10 cohort studies. Atherosclerosis. (2017) 262:51–4. doi: 10.1016/j.atherosclerosis.2017.05.007, PMID: 28511049

[22] Li K Sinclair AJ Zhao F Li D . Uncommon fatty acids and cardiometabolic health. Nutrients. (2018) 10:1559. doi: 10.3390/nu10101559, PMID: 30347833 PMC6213525

[23] Khaddaj-Mallat R Morin C Rousseau É . Novel n-3 PUFA monoacylglycerides of pharmacological and medicinal interest: Anti-inflammatory and anti-proliferative effects. Eur J Pharmacol. (2016) 792:70–7. doi: 10.1016/j.ejphar.2016.10.038, PMID: 27818127

[24] Tian Y Katsuki A Romanazzi D Miller MR Adams SL Miyashita K . Docosapentaenoic acid (22:5n-3) downregulates mRNA expression of pro-inflammatory factors in LPS-activated murine macrophage like RAW264.7 Cells. J Oleo Sci. (2017) 66:1149–56. doi: 10.5650/jos.ess17111, PMID: 28924088

[25] Del Gobbo LC Imamura F Aslibekyan S Marklund M Virtanen JK Wennberg M . Omega-3 polyunsaturated fatty acid biomarkers and coronary heart disease: pooling project of 19 cohort studies. JAMA Internal Med. (2016) 176:1155–66. doi: 10.1001/jamainternmed.2016.2925, PMID: 27357102 PMC5183535

[26] Byelashov OA Sinclair AJ Kaur G . Dietary sources, current intakes, and nutritional role of omega-3 docosapentaenoic acid. Lipid Technol. (2015) 27:79–82. doi: 10.1002/lite.201500013, PMID: 26097290 PMC4467567

[27] Moon SJ Jang HN Kim JH Moon MK . Lipid profiles in primary aldosteronism compared with essential hypertension: propensity-score matching study. Endocrinol Metab (Seoul). (2021) 36:885–94. doi: 10.3803/EnM.2021.1012, PMID: 34372626 PMC8419600

[28] Joint Committee for Guideline Revision . 2018 chinese guidelines for prevention and treatment of hypertension-A report of the revision committee of chinese guidelines for prevention and treatment of hypertension. J Geriatr Cardiol. (2019) 16:182–241. doi: 10.11909/j.issn.1671-5411.2019.03.014, PMID: 31080465 PMC6500570

[29] Chinese Society of Endocrinology . Expert consensus on the diagnosis and treatment of primary aldosteronism (2020). Chin J Endocrinol Metab. (2020) 36:727–36. doi: 10.3760/cma.j.cn311282-20200615-00444, PMID: 40668938

[30] Liu X Sun L Wen W Qiu M Luo J Li W . Association between the ratio of serum eicosapentaenoic acid to arachidonic acid and risk of coronary artery disease in young Chinese patients. Front Nutr. (2022) 9:1019058. doi: 10.3389/fnut.2022.1019058, PMID: 36407537 PMC9668899

[31] Joint Committee on the Chinese Guidelines for Lipid Management . Chinese guideline for lipid management (primary care version 2024). Chin J Cardiol. (2024) 52:330–7. doi: 10.3760/cma.j.cn112148-20240102-00002, PMID: 38548600

[32] Chinese Diabetes Society . Guideline for the prevention and treatment of diabetes mellitus in China (2024 edition). Chin J Diabetes Mellitus. (2025) 17:16–139. doi: 10.3760/cma.j.cn115791-20241203-00705, PMID: 40668938

[33] Department of Medical Administration National Health Commission of the People’s Republic of China . Chinese guidelines for the clinical management of obesity (2024 edition). Med J Peking Union Med Coll Hosp. (2025) 16:90–108. doi: 10.12290/xhyxzz.2024-0918

[34] Lang RM Badano LP Mor-Avi V Afilalo J Armstrong A Ernande L . Recommendations for cardiac chamber quantification by echocardiography in adults: an update from the American Society of Echocardiography and the European Association of Cardiovascular Imaging. J Am Soc Echocardiogr. (2015) 28:1–39.e14. doi: 10.1016/j.echo.2014.10.003, PMID: 25559473

[35] Adler GK Stowasser M Correa RR Khan N Kline G McGowan MJ . Primary aldosteronism: an endocrine society clinical practice guideline. J Clin Endocrinol Metab. (2025) 110:2453–95. doi: 10.1210/clinem/dgaf284, PMID: 40658480

[36] Sowers JR Whaley-Connell A Epstein M . Narrative review: the emerging clinical implications of the role of aldosterone in the metabolic syndrome and resistant hypertension. Ann Intern Med. (2009) 150:776–83. doi: 10.7326/0003-4819-150-11-200906020-00005, PMID: 19487712 PMC2824330

[37] Kurisu S Shimonaga T Iwasaki T Mitsuba N Ishibashi K Dohi Y . Mean platelet volume in patients with primary aldosteronism and its relation to left ventricular hypertrophy. Int J Cardiol. (2013) 168:3143–4. doi: 10.1016/j.ijcard.2013.04.156, PMID: 23664052

[38] Charlotte DCC van der Heijden MD Smeets EMM Aarntzen EHJG . Arterial wall inflammation and increased hematopoietic activity in patients with primary aldosteronism. J Clin Endocrinol Metab. (2020) 105:e1967–80. doi: 10.1210/clinem/dgz306, PMID: 31875423 PMC7105350

[39] Nys N Khatib AM Siegfried G . Apela promotes blood vessel regeneration and remodeling in zebrafish. Sci Rep. (2024) 14:3718. doi: 10.1038/s41598-023-50677-1, PMID: 38355946 PMC10867005

[40] Minakuchi H Wakino S Urai H Kurokochi A Hasegawa K Kanda T . The effect of aldosterone and aldosterone blockade on the progression of chronic kidney disease: a randomized placebo-controlled clinical trial. Sci Rep. (2020) 10:16626. doi: 10.1038/s41598-020-73638-4, PMID: 33024237 PMC7538950

[41] Nanba K Baker JE Blinder AR Bick NR Liu CJ Lim JS . Histopathology and genetic causes of primary aldosteronism in young adults. J Clin Endocrinol Metab. (2022) 107:2473–82. doi: 10.1210/clinem/dgac408, PMID: 35779252 PMC9761569

[42] Luther JM . Effects of aldosterone on insulin sensitivity and secretion. Steroids. (2014) 91:54–60. doi: 10.1016/j.steroids.2014.08.016, PMID: 25194457 PMC4252580

[43] Egalini F Guardamagna O Gaggero G Varaldo E Giannone B Beccuti G . The effects of omega 3 and omega 6 fatty acids on glucose metabolism: An updated review. Nutrients. (2023) 15:2672. doi: 10.3390/nu15122672, PMID: 37375575 PMC10301273

[44] Reincke M Fischer E Gerum S Merkle K Schulz S Pallauf A . Observational study mortality in treated primary aldosteronism: the German Conn’s registry. Hypertension. (2012) 60:618–24. doi: 10.1161/HYPERTENSIONAHA.112.197111, PMID: 22824982

[45] Alfhili MA Alsughayyir J Basudan A Alfaifi M Awan ZA Algethami MR . Blood indices of omega-3 and omega-6 polyunsaturated fatty acids are altered in hyperglycemia. Saudi J Biol Sci. (2023) 30:103577. doi: 10.1016/j.sjbs.2023.103577, PMID: 36816730 PMC9932443

[46] Markworth JF Kaur G Miller EG Larsen AE Sinclair AJ Rao Maddipati K . Divergent shifts in lipid mediator profile following supplementation with n-3 docosapentaenoic acid and eicosapentaenoic acid. FASEB J. (2016) 30:3714–25. doi: 10.1096/fj.201600360R, PMID: 27461565 PMC5067251

[47] Wu H He H Han T Tian X Zhu Z . Targeting cholesterol-dependent adrenal steroidogenesis for management of primary aldosteronism. Trends Endocrinol Metab. (2025) 36:789–801. doi: 10.1016/j.tem.2024.12.001, PMID: 39765399

[48] Murakami M . Fatty acid profiles in aldosterone-producing adenoma: Insights into pathogenetic significance. Hypertens Res. (2025) 48:2002–4. doi: 10.1038/s41440-025-02211-1, PMID: 40240872

[49] Miller E Kaur G Larsen A Loh SP Linderborg K Weisinger HS . A short-term n-3 DPA supplementation study in humans. Eur J Of Nutr. (2013) 52:895–904. doi: 10.1007/s00394-012-0396-3, PMID: 22729967

[50] Linderborg KM Kaur G Miller E Meikle PJ Larsen AE Weir JM . Postprandial metabolism of docosapentaenoic acid (DPA, 22:5n-3) and eicosapentaenoic acid (EPA, 20:5n-3) in humans. Prostaglandins Leukot Essent Fatty Acids. (2013) 88:313–9. doi: 10.1016/j.plefa.2013.01.010, PMID: 23433939

[51] Cheng SY Chen YJ Lin HC Chang HY Huang MD . Genetic analysis of polyunsaturated fatty acids biosynthesis pathway determines four distinct thraustochytrid types. Environ Microbiol. (2025) 27:e70090. doi: 10.1111/1462-2920.70090, PMID: 40152028 PMC11951076

[52] Zhang Y Zhang M Lyu B Kishi H Kobayashi S . Omega-3 and omega-6 DPA equally inhibit the sphingosylphosphorylcholine-induced Ca(2+)-sensitization of vascular smooth muscle contraction via inhibiting Rho-kinase activation and translocation. Sci Rep. (2017) 7:36368. doi: 10.1038/srep36368, PMID: 28169288 PMC5294466

[53] Morin C Hiram R Rousseau E Blier PU Fortin S . Docosapentaenoic acid monoacylglyceride reduces inflammation and vascular remodeling in experimental pulmonary hypertension. Am J Physiol Heart Circ Physiol. (2014) 307:H574–86. doi: 10.1152/ajpheart.00814.2013, PMID: 24929859

[54] Heydari B Abdullah S Pottala JV Shah R Abbasi S Mandry D . Effect of omega-3 acid ethyl esters on left ventricular remodeling after acute myocardial infarction: the OMEGA-REMODEL randomized clinical trial. Circulation. (2016) 134:378–91. doi: 10.1161/CIRCULATIONAHA.115.019949, PMID: 27482002 PMC4973577

