# Supplementary material for: DPA/AA ratio as a potential biomarker of primary aldosteronism in young and middle-aged adults
Source: Front Endocrinol (Lausanne). 2026 Mar 11;17:1771955. doi: 10.3389/fendo.2026.1771955 (PMC13012993; doi:10.3389/fendo.2026.1771955)
Supplement: Supplementary file 1 [file DataSheet1.docx]

**Supplementary Materials**

**Measurement of the serum levels of PUFAs**

The serum samples of the enrolled patients in this study were sent to the Dalian Institute of Chemical Physics for the determination of serum free fatty acids using isotope dilution-liquid chromatography-tandem mass spectrometry (AB SCIEX Triple Quad 4500MD LC-MS/MS).

Deuterated free fatty acids were used as the isotope internal standards. After addition to serum samples, pretreatment procedures including protein precipitation, organic solvent extraction, nitrogen blow-drying, and reconstitution were performed to remove interfering substances such as proteins and phospholipids from the serum matrix. The target free fatty acids (FFAs) and internal standards were separated by reversed-phase liquid chromatography. Detection was carried out using an AB SCIEX Triple Quad 4500MD mass spectrometer equipped with an electrospray negative ionization source (ESI⁻) in multiple reaction monitoring (MRM) mode. The isotope internal standards were applied to correct for losses during pretreatment and matrix effects. The actual concentrations of individual FFAs in serum were calculated from the peak area ratios of the analytes to their corresponding internal standards, combined with the standard calibration curve. This method, namely isotope dilution-liquid chromatography-tandem mass spectrometry (ID-LC-MS/MS), ensures the accuracy and reliability of quantitative results.

All operations were performed in an ice bath to prevent FFA degradation, and operational consistency was strictly controlled to minimize experimental errors. A 1.5 mL centrifuge tube was taken, and 50 μL of serum sample was added. Then 50 μL of internal standard working solution (1 μg/mL) was added to each tube, followed by vortex mixing for 30 s to achieve thorough mixing of the internal standard with serum (final internal standard concentration: 0.5 μg/mL). 150 μL of acetonitrile was added, and the mixture was vortexed for 1 min at 2500 r/min, then allowed to stand in an ice bath for 5 min to fully precipitate proteins in the serum. The centrifuge tube was placed in a high-speed refrigerated centrifuge and centrifuged at 4 °C and 12000 r/min for 10 min.

The supernatant was transferred to a new 1.5 mL centrifuge tube. The supernatant was dried under a nitrogen evaporator at 37 °C with a nitrogen flow rate of 5 mL/min until no liquid residue remained. 100 μL of initial mobile phase (water + 0.01% formic acid + 0.2 mM ammonium formate: acetonitrile + 0.01% formic acid = 95:5, v/v) was added to the dried tube, followed by vortex mixing for 1 min to fully reconstitute the residue. The reconstituted solution was filtered through a 0.22 μm PTFE syringe filter, and the filtrate was collected in an autosampler vial for LC‑MS/MS analysis.

**Supplementary Results**

Suppl Table 1 ω-3, ω-6 PUFAs and ratios of ω-3/ω-6 PUFAs of PA in young and middle-aged patients

| Fatty acids  (nmol/mL) | Patients with EH  (*n* = 138) | Patients with PA  (*n* = 138) | *P* value |
| --- | --- | --- | --- |
| EPA | 92.00 (54.00–168.00) | 98.50 (66.00–165.75) | 0.480 |
| DHA | 353.00 (262.25–470.75) | 419.00 (281.50–553.50) | 0.020 |
| DPA | 102.00 (70.00–132.75) | 88.50 (68.00–114.75) | 0.060 |
| AA | 1091.50 (853.00–1492.00) | 1381.00 (1118.75–1731.50) | <0.001 |
| EPA + DHA | 454.50 (332.75–647.75) | 515.00 (359.00–734.25) | 0.050 |
| EPA + DPA | 187.00 (146.00–308.75) | 194.50 (140.25–271.25) | 0.830 |
| DHA + DPA | 442.50 (354.00–623.50) | 521.00 (357.00–666.50) | 0.120 |
| EPA + DHA + DPA | 541.00 (416.25–787.00) | 622.00 (435.50–820.25) | 0.160 |
| EPA/AA | 0.08 (0.05–0.15) | 0.07 (0.05–0.10) | 0.120 |
| DHA/AA | 0.32 (0.24–0.45) | 0.31 (0.23–0.40) | 0.090 |
| DPA/AA | 0.08 (0.06–0.14) | 0.06 (0.05–0.08) | <0.001 |
| (EPA + DHA)/AA | 0.42 (0.30–0.58) | 0.40 (0.29–0.49) | 0.070 |
| (EPA + DPA)/AA | 0.18 (0.12–0.26) | 0.15 (0.11–0.19) | <0.001 |
| (DHA + DPA)/AA | 0.42 (0.32–0.54) | 0.38 (0.29–0.48) | 0.010 |
| (EPA + DHA + DPA)/AA | 0.52 (0.39–0.70) | 0.46 (0.35–0.59) | 0.010 |

Data are presented as percentages, mean and SD, and median and interquartile range. AA, arachidonic acid; DHA, docosahexaenoic acid; DPA, docosapentaenoic acid; DHA/AA, ratio of DHA and AA; DPA/AA, ratio of DPA and AA; EPA, eicosapentaenoic acid; EPA/AA, ratio of EPA and AA; (EPA + DHA)/AA, ratio of EPA and DHA to AA; (EPA + DPA)/AA, ratio of EPA and DPA to AA; (DHA + DPA)/AA, ratio of DHA and DPA to AA; (EPA + DPA + DHA)/AA, ratio of EPA, DPA, and DHA to AA.

Suppl Table 2 Multivariate regression analysis of the traditional risk factors of PA in young and middle-aged patients

|  | OR (95% CI) | *P* |  | OR (95% CI) | *P* |  |  |  |
| --- | --- | --- | --- | --- | --- | --- | --- | --- |
| Model 1 |  |  | Model 2 |  | | |  |  |
| SBP | 1.020(1.006–1.035) | 0.006 | DBP | 1.033 (1.008–1.058) | 0.009 |  |  |  |
| FBG | 1.312 (1.046–1.647) | 0.019 | PLT | 0.994 (0.989–1.000) | 0.044 |  |  |  |
| PLT | 0.994 (0.989–0.999) | 0.024 | LDL-C | 1.858 (1.127–3.064) | 0.015 |  |  |  |
| LDL-C | 2.064 (1.20–3.382) | 0.004 | AST | 0.964 (0.935–0.994) | 0.020 |  |  |  |
| AST | 0.962 (0.934–0.991) | 0.010 | Blood potassium | 0.060 (0.026–0.135) | <0.001 |  |  |  |
| Blood potassium | 0.094 (0.046–0.190) | <0.001 | UACR | 1.004 (1.001–1.007) | 0.002 |  |  |  |

Model 1 included SBP, DBP, blood potassium, blood sodium, PLT, FBG, ALT, AST, LDL-C, and UA; Model 2 included the variables of Model 1 and left ventricular hypertrophy and UACR.


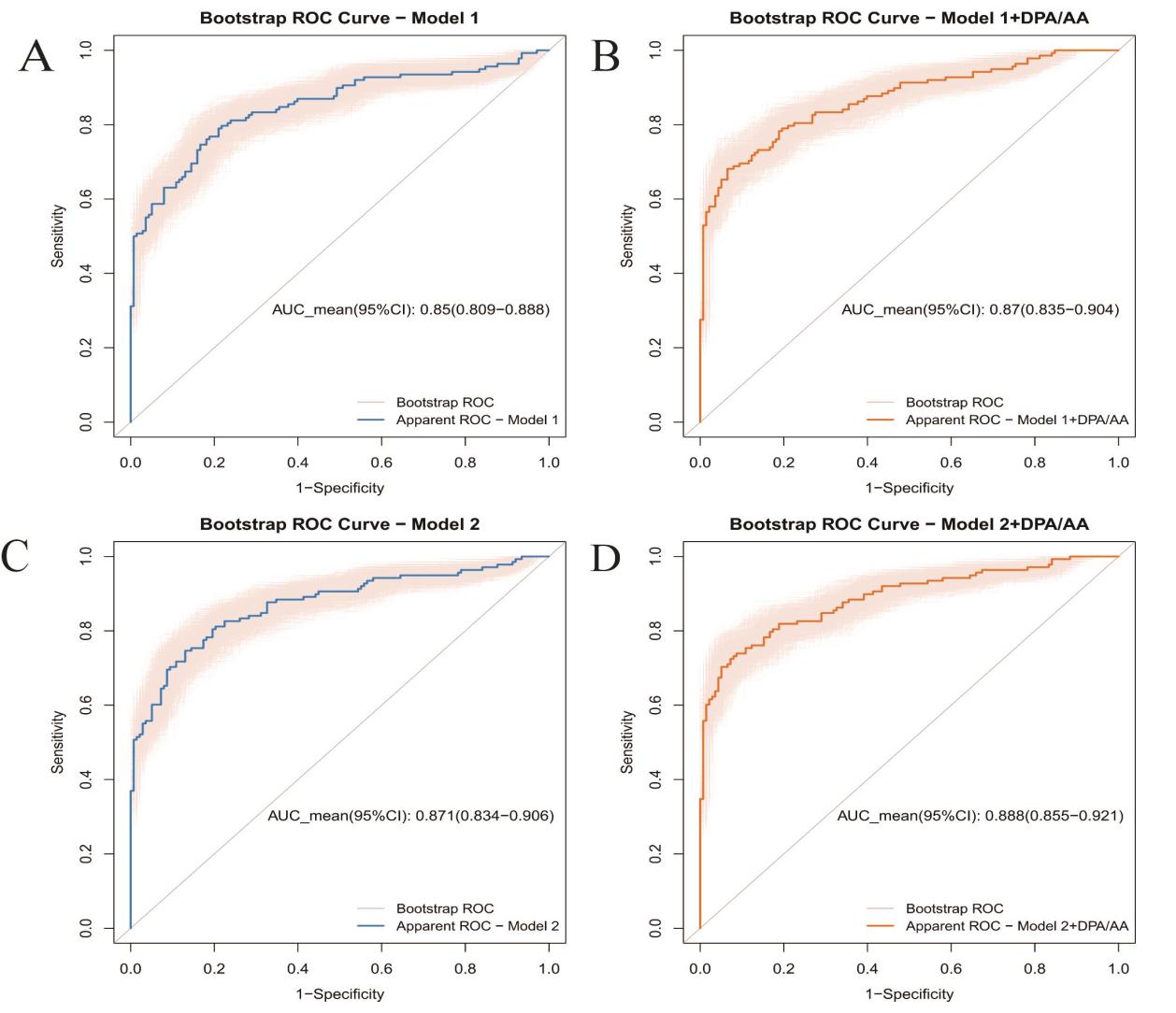


Suppl Figure 1 **Internal** v**alidation of** m**odel 1 and** m**odel 2** w**ith and** w**ithout DPA/AA Ratio via the Bootstrap Method**. A. Bootstrap ROC of Model 1; B. Bootstrap ROC of Model 1+DPA/AA; C. Bootstrap ROC of Model 2; D. Bootstrap ROC of Model 2+DPA/AA


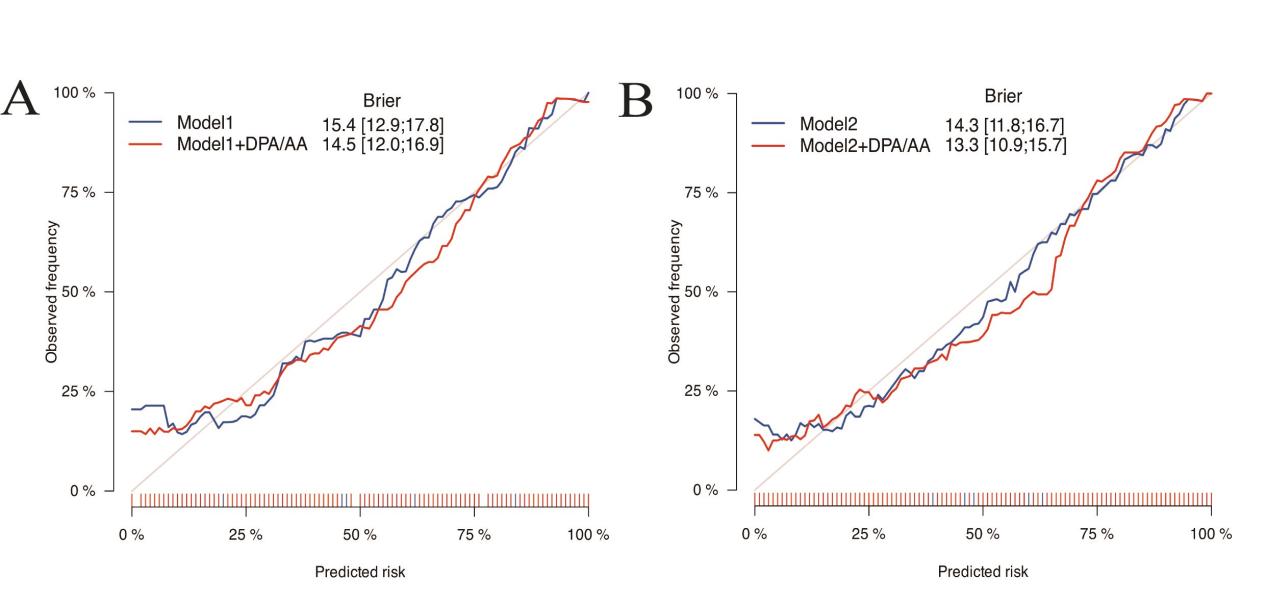


Suppl Figure 2 Calibration plots of PA prediction models with and without PUFA indices in Young and Middle-aged Patients

A. Calibration plots of Model 1 and Model 1+DPA/AA; B. Calibration plots of Model 2 and Model 2+DPA/AA


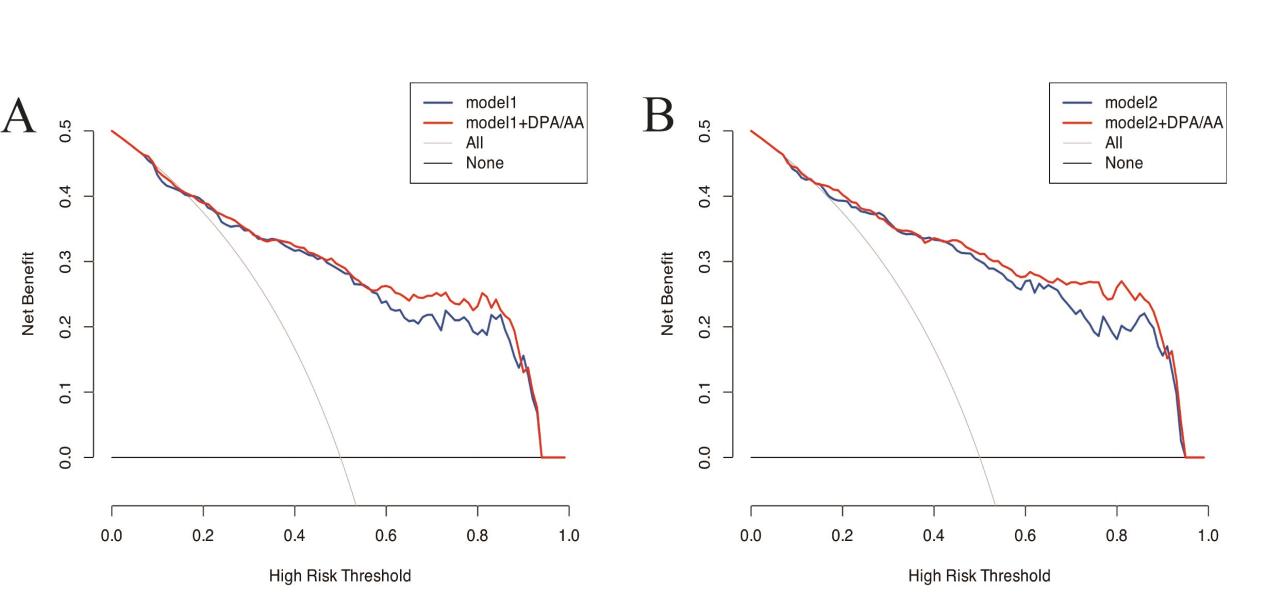


Suppl Figure 3 Decision Curve Analysis of PA Prediction Models With and Without PUFA Ratios in Young and Middle-aged Patients. A. DCA curve of Model1 and Model1+DPA/AA; B. DCA curve of Model 2 and Model2+DPA/AA

Bootstrap validation verified the robustness of all models: Model 1 yielded a bootstrap AUC of 0.85 (95% CI: 0.809–0.888), Model 1+DPA/AA of 0.87 (95% CI: 0.835–0.904), Model 2 of 0.871 (95% CI: 0.834–0.906), and Model 2+DPA/AA of 0.888 (95% CI: 0.855–0.921) (Suppl Figure 1). Calibration curves demonstrated satisfactory calibration for all developed models (Suppl Figure 2), and decision curve analysis (DCA) confirmed their favorable clinical utility (Suppl Figure 3).
